# Does descending health resources reform impact patient low-level hospital selection behavior? Evidence from Zhejiang, China

**DOI:** 10.1186/s13690-021-00700-6

**Published:** 2021-10-18

**Authors:** Zesheng SUN, Shuhong WANG, Hongjun ZHAO, Xu ZHOU, Ludan ZHANG, Jiongping SHI

**Affiliations:** 1 School of Finance and Business, Shanghai Normal University, Shanghai, China; 2 Department of Stomatology, Tongde Hospital of Zhejiang Province, Hangzhou, China; 3 Department of Stomatology, Songjiang Hospital of Shanghai, Shanghai, China; 4 School of Finance, Zhejiang Gongshang University, Hangzhou, China

**Keywords:** Descending resources reform, Low-level hospitals, Patient satisfaction, Consumer behavior, Hospital selection behavior

## Abstract

**Background:**

Since 2013, China launched descending resources reform, which is a new attempt to correct unbalanced allocation of health resources through human capital spillovers and brand implantation from high-level hospitals. The purpose of this paper is to explore the patients’ hospital selection response to this reform with the focus of low-level hospitals to better understand the effect of this reform on correcting regional inequality of health resources allocation.

**Methods:**

The European Consumer Satisfaction Index model (ECSI) was used to design a questionnaire, and cross-sectional data from 17 hospitals were collected through 1287 questionnaires from Zhejiang Province. Patient hospital selection (loyalty) is measured using ordinary variables by considering patient willingness to choose a low-level hospital when suffering an illness or severe illness. Analysis of variance (ANOVA) and the structure equation model are applied to examine the effect of reforms on patient behavior.

**Results:**

The descending resources reform promotes improvements in the capabilities and medical environment of low-level hospitals, and descending doctors also have high accessibility. Perceived quality, patient expectations, and hospital image have significant positive effects on patient satisfaction, and the explanatory power of brand implantation from cooperative high-level hospitals and descending doctors is stronger than the image of the low-level hospital itself. And descending resources reform and patient satisfaction have significant positive impacts on patient’s choice for low-level hospitals with the existence of mediating effect of satisfaction.

**Conclusions:**

This paper provides supporting empirical evidence of the descending resources reform’s impact on patients’ low-level hospital selection. This reform has been effective in improving the capabilities of low-level hospitals, and brand implantation of high-level hospitals shows strong explanatory power. China’s reform offers a distinct and valuable approach to correcting the uneven allocation of health resources. Besides, the findings also suggest that policymakers could pay more attention to the importance of information channels in impacting patient awareness, responses, and hospital selection.

## Background

Regional inequality of health resources allocation is a global concern, and a key focus is access issues between urban and rural areas [1]. Rural areas typically face the challenge of doctor shortages due to the difficulties with transport and communications that exist in most developing and developed countries [2]. China faces a different constraint in terms of its structural congestion between overcrowded (city) high-level hospitals and idle (county and town) low-level hospitals due to patients’ biased behavior, which motivates them to choose high-level hospitals [3]. This dynamic stems from China’s long-lasting price regulation and health resource concentration in (city) high-level hospitals, and it generates medical cost and efficiency losses as well as doctor–patient conflicts and detrimental social consequences [4]. Since 2003, following the SARS crisis, the Chinese government has paid increasing attention to investment in the infrastructure of (mainly town) low-level hospitals. Policies such as higher government health expenditure, expansion of medical insurance coverage, and abolition of marked-up drug prices have been implemented since 2009. However, increasing health care affordability due to expanded medical insurance coverage only worsened the structural congestion [5, 6]; the efficiency of low-level hospitals has yet not improved [7].

In China, the dominant public hospital system divides hospitals into three different levels, from first to third. All hospitals run by MOH (Ministry of Health of China) and nearly all hospitals run by provinces and major cities were approved to be ranked at the third level, and are thus viewed as high-level hospitals. Community and township hospitals were identified as first level, and district- or county-level hospitals were generally approved to be second level; these hospitals are usually viewed as low-level hospitals with community and township ones being the lowest.

Previously, the guiding concept and perception was that low-level hospitals could only diagnose and treat a limited range of ailments and offer a lower standard of care than high-level hospitals. Presently, underutilization and the low capabilities of low-level hospitals are still severe challenges for China [8]. These issues reflect the fact that previous investment focused on fixed assets rather than human capital [3]. This kept low-level hospitals at a disadvantage in attracting patients due to the differences in human capital between different levels of hospitals coupled with regulated medical service prices. The uneven allocation of health resources accelerated after 2009 due to reforms that regulated prices and expanded insurance coverage, which then enabled more patients to afford high-level hospitals [9].

This history indicates that past demand-side and supply-side reforms were unsuccessful in realizing regional equality. One new solution for this problem is the descending resources reform, introduced in Zhejiang and other provinces in China in 2013. The core idea of this reform is to encourage high-level hospitals to establish cooperative ties with low-level hospitals, thus driving the high-level hospitals’ human capital to transfer down (i.e., descend) to the low-level hospitals. As part of this policy, the government would provide a subsidy to (at least partially) compensate hospitals for the reform costs. Overall, the policy aims are to (1) narrow the human capital gaps among hospitals via spillover effects, and (2) imbed (brand) the image of a high-level hospital on a low-level one, which could then help reshape patients’ behavior in terms of hospital selection, with an emphasis on low-level hospitals [10]. This reform makes full use of the dominant role of China’s public hospital system, but there is still little empirical evidence on this reform’s effect on patients’ care-provider choices. This paper explores this issue by using a structural equation model (SEM) based on questionnaire data collected in Zhejiang, China.

Patient hospital choices can be viewed as reflecting patient loyalty to different care providers, and in the context of China’s public hospital system, these choices can be viewed as patient loyalty to different levels of hospital. Different from the traditional literature on patient satisfaction [11, 12],^1^ this paper uses the term “*patient satisfaction with the reform*” to measure patients’ response for the reform and its impact on low-level hospitals. This satisfaction would thus reflect patients’ loyalty to and thus their choice behavior in terms of choosing low-level hospitals.

In the marketing science literature, a satisfaction index model can be used to discuss a reform’s effect on patient satisfaction and loyalty. The Swedish Customer Satisfaction Barometer (SCSB model) emphasizes the determinants of two antecedent factors: customer expectations and perceived performance; customer satisfaction then affects customer complaints and ultimately impacts customer loyalty [13]. The American Consumer Satisfaction model (ASCI) proposed by Fornell et al. [14] adds the latent variable of perceived quality, but still uses perceived value to measure perceived performance. Brady and Cronin [15] emphasized service quality evaluations based on the dimensions of results, interaction quality, and physical environment quality, similar to the ACSI model.

The European Consumer Satisfaction Model (ECSI) initiated by the European Commission in 1999 removed the latent variable of customer complaint from the ACSI and SCSB models because complaint processing has no significant impact on customer satisfaction or loyalty in empirical research [16]. The ECSI model includes corporate image, a move intended to incorporate customers’ memory associated with organizations [17]; and satisfaction mediates between service quality and loyalty [18, 19]. Meanwhile, some literature has also highlighted the impact of demographic and exogenous policy variables on satisfaction [20, 21]. However, there are few empirical studies on how health policies affect patient satisfaction and loyalty.

Relevant studies in China mainly discussed the impacts of the circa-2009 health reforms on the efficiency of low-level hospitals, but these studies did not use micro-data collected at the individual level, nor were they involved the descending resources reform [6]. Some studies used micro-data to explore patient satisfaction in developing countries including China using demographic variables including age, gender, and education level [22, 23]; other studies used medical market concentration, income, and health insurance status [24, 25], as well as other factors. Researches on developing countries also utilized scales to evaluate the relationships among service quality, satisfaction, and loyalty [26, 27]. However, these studies treated the institutional environment as given, an assumption that is inconsistent with the circumstances in developing countries experiencing a rapidly evolving healthcare system and reforms.

Different from reforms in developing countries, which focus on designing different health resource formulae and financing mechanisms [28], China’s descending resources reform is paving a new way to correct the uneven allocation of health resources. Some recent studies discussed the impact of this reform on both doctors or patients [25, 29], but the OLS/OLM (ordinary least square or ordered logit model) methodology used by Sun et al. [25] could not solve measurement error of survey data. The contribution of this paper is to explore this reform’s effect on patient hospital-selection behavior using a SEM model. Health policy is incorporated into the patient response model in order to better understand the effect of the descending resources reform. Meanwhile, different levels of hospitals and cognitive channels are introduced to discuss the heterogeneous effects of the reform on patient behavior.

## Materials and methods

The European Consumer Satisfaction Index model is used as the basic model for this study, and two exogenous variables are included: the descending resource reform and demographics. We use loyalty to a low-level hospital (LLH) to measure patients’ hospital choice, which is affected by patient satisfaction [19, 30] and two exogenous variables. Satisfaction is affected by three latent variables: perceived quality, consumer expectations, and hospital image. According to the ECSI model, the difference between consumer expectations and perceived performance is expressed as the expected value, but technical reliability and treatment effects of medical services are difficult for patients to evaluate [31], so the perceived value cannot be directly observed. Donabedian [32] suggested using other non-technical variables such as convenience and information; however, these suggested variables are already included in perceived quality and demographics.

The descending resources reform can affect patient satisfaction in two ways. First, it substantially changes low-level hospitals’ capabilities, which have been included in the latent variables, as well as impacting perceived quality and hospital image. Second, the reform information can be transmitted to patients and impact those patients’ choices. Latent variables of the reform include (1) whether the reform information is correctly recognized by patients, (2) the information channel, and (3) related policy on medical service price, differential medical insurance reimbursement and tiered medical services, which are derived from individual hospitals.

### Questionnaire design and data

The question items and their definitions of latent variable are reported in Table 1, where a five-point unbalanced scale is used for ordered variables. Patient hospital choice (loyalty) is measured as *the intention to choose a local low-level hospital* and *the intention to choose a local low-level hospital first when suffering a serious illness*, respectively. Latent variables of patient satisfaction include (1) reform satisfaction at the industry level, and (2) satisfaction with local low-level hospitals. For the three exogenous latent variables of the ESCI model, hospital image refers to patients’ brand recognition perception of the hospital, and patient trust provides the basis of future cooperation in terms of patients’ future hospital choices [33]. In addition, patients’ awareness of the descending (high-level) hospital and descending doctors provide a measurement of the degree to which the image of high-level hospitals is implanted onto low-level hospitals. The aforementioned three variables are used as measurement variables of hospital image.

**Table 1:** Questionnaire scale, variables, and definitions

Variable types	Latent variables		Measurement variables		
	Symbol	Name	Symbol	Question items	Definition
Exogenous variables	ξ1	Hospital image	X11	Trust for LLH	1 for negative change, 2 for no change, 3–5 for ordinary positive change
			X12	Awareness for the descending hospitals	1–5 ordinary variables from very low to very high
			X13	Awareness for the descending doctors	1–5 ordinary variables from very low to very high
	ξ2	Patient expectation	X21	Accessibility to the descending doctors	1–5 ordinary variables from very low to very high
			X22	LLH capability change	1 for negative change, 2 for no change, 3–5 for ordinary positive change
			X23	Medical cost change	1 for positive change, 2 for no change, 3–5 for ordinary negative change
	ξ3	Perceived quality	X31	Convenience changes	1–5 ordinary variables from very low to very high
			X32	LLH Environment change	1–5 ordinary variables from very low to very high
	ξ4	Reform policy	X41	Reform Recognition	1–5 ordinary variables from very low to very high
			X42	Reform information Channels	1 for public channels (newspaper, TV and hospital), private channels being 0
			X43	Medical service price	1–5 ordinary variables from very low to very high
			X44	Insurance reimbursement	1–5 ordinary variables from very low to very high
			X45	Tiered medical service	1–5 ordinary variables from very low to very high
	ξ5	Socio-demographics	X51	Gender	1 for male and 0 for female
			X52	Age	1 for ≤30, 2–5 for 31–40, 41–50, 51–60, and ≥ 61, respectively
			X53	Education level	1 for primary or below, 2–5 for junior, high school, college or university, graduate degree, respectively
Mediating variable	ξ6	Satisfaction	ME1	Reform satisfaction	1–5 ordinary variables from very low to very high
			ME2	LLH satisfaction	1–5 ordinary variables from very low to very high
Endogenous variable	η	Hospital selection (Loyalty)	Y1	Intention to choose LLH	1–5 ordinary variable from very low to very high
			Y2	Intention to choose LLH when suffering serious illness	1–5 ordinary variables from very low to very high

Source: The authors

Clavolino and Dalsgaard [34] pointed out that patient expectations are related to prior expectations of the observed existing services. Because the descending resources reform involves both high-level and low-level hospitals, we take the diagnosis/treatment capability of low-level hospitals and the accessibility of descending doctors as measurement variables. Patient expectations are also related to medical costs, which are included as a measurement variable of patient expectations. The latent variables of perceived quality are related to associated services, and we further considered the medical environment and convenience of low-level hospitals as measurement variables.

Socio-demographic variables include gender, age, and education level. Awareness of reforms, cognition channels, and reform-related policy evaluations regarding medical service prices, differentiated insurance reimbursements, and tiered medical services are incorporated to measure the latent variable of reform. Of these, differentiated insurance reimbursement policies will incentivize patients to choose low-level hospitals by reducing/increasing the reimbursement ratio when choosing high- or low-level hospitals, respectively. The tiered medical services policy requires patients to choose a low-level hospital first, and then be referred to a high-level hospital if suffering a serious illness, which impacts patients’ satisfaction and hospital choice behavior.

The data used in this study cover 17 public hospitals in Zhejiang Province, China, including six tertiary hospitals, eight secondary hospitals, and three primary hospitals. In each hospital, face-to-face interviews were performed in the outpatient department, where questionnaires were randomly distributed to patients by trained independent investigators. All interviewees that finished the questionnaire received a small gift worth $1.00 (7 RMB Yuan) for their time. From November 2018 to October 2019, we collected 1354 questionnaires, among which 1287 were valid, an effective rate of 95.05%.

### Empirical methods

The samples used in this paper cover three different levels of hospitals and different information channels, so we first use one-way analysis of variance (ANOVA) to examine whether significant differences exist among patients at different levels of hospitals and information channels. If the ANOVA results (F-test) reach the threshold value (α = 0.05), a significant difference exists. Then, a multiple posteriori comparison will be performed to compare the differences by using the LSD (least significant difference) test.

Because the data used in this paper come from questionnaires, patients’ cognitions of and responses to the reform are subjective and difficult to directly measure, meaning it is hard to avoid subjective measurement errors. The structural equation model (SEM) has the advantage of handling multiple variables and measurement errors of variables. In addition, this method can estimate both the factor structure and factor relationships, which makes it suitable to process and analysis questionnaire data. According to Qiu and Lin [35], this model consists of a measurement equation and structure equation as follows:

1 $$ Y={\Lambda}_y\eta +\varepsilon $$

2 $$ X={\Lambda}_x\xi +\delta $$

3 $$ \eta = B\eta +\Gamma \xi +\zeta $$

Equations (1) and (2) are measurement equations to describe the relationship between latent variables and measurement variables. *Y* and *X* are the observable variables of endogenous and exogenous latent variables respectively, *η* and *ξ* are endogenous and exogenous latent variables respectively, and Λ_*y*_ and Λ_*x*_ are the factor loading matrix. Equation (3) provides the structure model, which is used to describe the relationship between latent variables, where in the structure coefficient matrix, *B* and Γ represent the relationship between endogenous latent variables and the impact of exogenous latent variables on endogenous latent variables, respectively; and *ζ* is the residual term matrix.

**Fig. 1:**
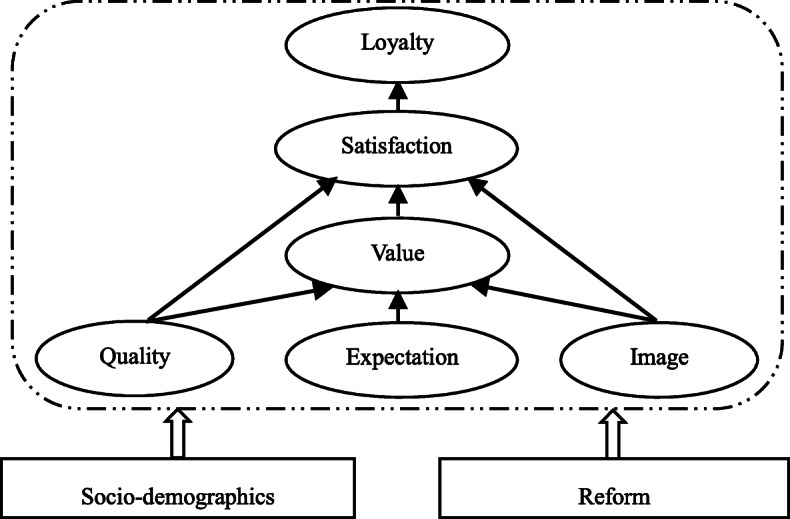
The theoretical model of patient hospital choice (loyalty). Source: The ESCI model is shown inside the dotted line, this model referred to Ref. [25]

Following the ESCI model, we establish a SEM model incorporating descending resources reform and hospital selection (Fig. 1). Detailed variables are given in Table 1. The empirical analysis is performed using two steps: (1) estimating the impact of different exogenous latent variables on patient satisfaction; and (2) exploring the impact of patient satisfaction and the reform on patients’ hospital selection (loyalty). In addition, subsamples of different levels of hospitals will be discussed to test the robustness of the results.

**Fig. 2:**
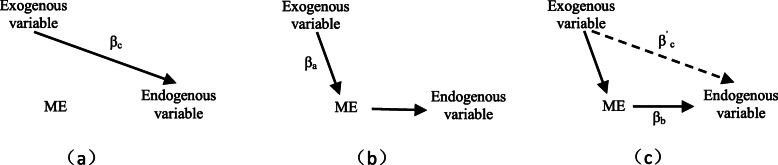
The estimation method of mediating effects. Sources: Baron and Kenny (1986)

Finally, in order to measure the existence of a mediating effect, referring to Baron and Kenny [36], we estimate *β*_*c*_, *β*_*a*_, *β*_*b*_, and *β*^*’*^_*c*_ shown in Fig. 2 in turn. If *β*^*’*^_*c*_ is insignificant and the others are significant, then complete mediating effects exist; however, if the estimated value of *β*^*’*^_*c*_ is significant and its absolute value is less than that of *β*_*c*_, then there is a partial mediating effect.

## Results

We used SPSS 23.0 software and reliability tests to assess the reliability and consistency of the scale and data. The results show that the Cronbach’s *α* coefficient is 0.915(> 0.800), indicating that the scales and data have a good internal consistency. Then, factor analysis was utilized to test the validity of the questionnaire. The KMO (Kaiser-Meyer-Olkin) value of 0.901 and Bartlett spheroid test value of 9223.199 (Sig. = 0.0001) show that the questionnaire has good structural validity, so the scale and data are suitable in performing empirical estimations. Using AMOS 21.0 software, the absolute fit indices of patient loyalty model shows that RMSEA (Root Mean Square Error of Approximation) =0.067(< 0.10), GFI (Goodness of Fit Indices) =0.934(> 0.90), AGFI (Adjusted Goodness of Fit) =0.846(> 0.80), and PGFI (Parsimony Goodness of Fit Indices) =0.545(> 0.50), suggesting that the data can be used for SEM model estimation.

### ANOVA and multiple comparison results

Table 2 presents the summary statistics for the sample variables. It can be seen that women account for 58% of the sample. The average age is between 2= “31-40 years old” and 3= “41-50 years old,” with a mean of (2.35 ± 1.25). The average education level is close to 3 = “high school” (2.88 ± 1.11). The mean reform satisfaction is (2.70 ± 1.39), which is between 2 = “low” and 3 = “fair”. However, satisfaction with local low-level hospitals reached (3.35 ± 0.87), where 87% of interviewees responded “positive,” “high,” and “very high,” 10% reported unchanged satisfaction levels, and only 1% reported that their satisfaction had declined. As for the hospital selection (loyalty) variable, the average score for “intention to choose local low-level hospital” is 3.30, and the loyalty score when suffering a serious illness reached 3.20 (3 = “positive” and 4 = “high”). Both of these results indicate that following the descending resources reform, patients will prefer to choose a low-level hospital.

**Table 2:** Descriptive statistics of key variables, ANOVA, and multiple comparison results

Latent variables	Measured variables	Overall	Hospital level			Information channel	
			Tertiary	Secondary	Primary	Public	Private
Hospital Image	X11	3.28 ± 0.98	3.29 ± 0.95	3.34 ± 1.01^*,1^	3.11 ± 0.87^*,1^	3.49 ± 0.94^*,1^	2.93 ± 0.95^*,1^
	X12	2.55 ± 1.26	3.05 ± 1.40^*,2^	2.49 ± 1.29^*,1^	2.48 ± 1.06^*,1^	2.98 ± 1.18^*,1^	1.78 ± 1.02^*,1^
	X13	2.50 ± 1.25	3.02 ± 1.46^*,2^	2.41 ± 1.27^*,1^	2.55 ± 1.02^*,1^	2.93 ± 1.18^*,1^	1.75 ± 0.99^*,1^
Patient expectation	X21	3.10 ± 1.10	2.96 ± 1.14	3.16 ± 1.14^*,1^	2.97 ± 0.96^*,1^	3.35 ± 1.01^*,1^	2.65 ± 1.11^*,1^
	X22	3.35 ± 0.85	3.28 ± 0.90	3.41 ± 0.86^*,1^	3.22 ± 0.78^*,1^	3.48 ± 0.83^*,1^	3.12 ± 0.84^*,1^
	X23	2.80 ± 0.99	2.88 ± 1.07	2.78 ± 0.99	2.80 ± 0.94	2.95 ± 1.02^*,1^	2.52 ± 0.86^*,1^
Perceived quality	X31	3.27 ± 1.00	3.29 ± 0.98	3.33 ± 1.03^*,1^	3.10 ± 0.92^*,1^	3.48 ± 0.96^*,1^	2.91 ± 0.97^*,1^
	X32	3.38 ± 0.91	3.35 ± 0.94	3.37 ± 0.92	3.41 ± 0.85	3.53 ± 0.89^*,1^	3.10 ± 0.87^*,1^
Reform policy	X41	2.36 ± 1.29	2.90 ± 1.44^*,2^	2.30 ± 1.32^*,1^	2.30 ± 1.09^*,1^	2.78 ± 1.27^*^	1.63 ± 0.96^*^
	X42	0.64 ± 0.48	0.72 ± 0.45	0.62 ± 0.49	0.65 ± 0.48	–	–
	X43	3.30 ± 0.87	2.99 ± 0.94^*,2^	3.35 ± 0.86^*,1^	3.30 ± 0.87^*,1^	3.46 ± 0.87^*,1^	3.02 ± 0.81^*,1^
	X44	3.20 ± 0.86	2.97 ± 0.93^*,2^	3.25 ± 0.87^*,1^	3.16 ± 0.80^*,1^	3.34 ± 0.86^*,1^	2.95 ± 0.80^*,1^
	X45	3.29 ± 0.90	3.15 ± 0.99^*,1^	3.35 ± 0.88^*,2^	3.20 ± 0.89^*,1^	3.41 ± 0.91^*,1^	3.08 ± 0.84^*,1^
Socio-demographics	X51	0.42 ± 0.49	0.32 ± 0.47^*,1^	0.45 ± 0.50^*,1^	0.39 ± 0.49	0.43 ± 0.50	0.40 ± 0.49
	X52	2.35 ± 1.25	2.41 ± 1.11	2.34 ± 1.28	2.34 ± 1.21	2.38 ± 1.24	2.29 ± 1.26
	X53	2.88 ± 1.11	3.34 ± 1.05^*,2^	2.81 ± 1.09^*,1^	2.87 ± 1.13^*,1^	2.89 ± 1.11	2.87 ± 1.10^*^
Satisfaction	ME1	2.70 ± 1.39	3.26 ± 1.49^*,2^	2.65 ± 1.42^*,1^	2.59 ± 1.19^*,1^	3.22 ± 1.24^*,1^	1.79 ± 1.15^*,1^
	ME2	3.35 ± 0.87	3.29 ± 0.88	3.40 ± 0.88^*,1^	3.23 ± 0.81^*,1^	3.50 ± 0.84^*,1^	3.08 ± 0.84^*,1^
Loyalty	Y1	3.30 ± 0.90	3.29 ± 0.88	3.36 ± 0.91^*,1^	3.13 ± 0.84^*,1^	3.43 ± 0.89^*,1^	3.08 ± 0.86^*,1^
	Y2	3.20 ± 0.96	3.42 ± 1.01^*,1^	3.26 ± 0.99^*,1^	2.94 ± 0.82^*,2^	3.33 ± 0.97^*,1^	2.97 ± 0.91^*,1^
Sample size		1287	1287	130	851	306	819

Note: [1] Asterisks and *n* = 1 or 2 (*, n) denote a statistically significant difference among the different hospital groups with ANOVA (α =0.05), and the number of differences by using multiple posteriori comparison

For measurement variables, trust in local low-level hospitals is (3.28 ± 0.98), showing that the descending resources reform has improved the image of low-level hospitals. However, respondents reported average scores between 2 = “low” and 3 = “fair” for awareness of descending high-level hospitals/doctors. In contrast, accessibility of the descending doctors reached (3.10 ± 1.10, where 3 = “positive” and 4 = “high”), indicating that respondents found the descending doctors easy to access. The variables of environment, capability, and convenience for local low-level hospitals all have means between 3.2 and 3.4 (3 = “positive” and 4 = “high”), suggesting that the reform had a positive impact from the patients’ perspective. In addition, the response for medical cost is (2.80 ± 0.99, where 2 = “no change” and 3 = “slight decrease”), indicating that the descending resources reform has lowered medical costs in general.

For the reform policy latent variable, 64% of patients obtain information from public channels such as newspapers, television, and the hospital, with a mean of (2.36 ± 1.29, where 2 = “low” and 3 = “fair”). However, reform-related policies like medical service prices, (differential) insurance reimbursement levels, and tiered medical services are evaluated high, with an average of 3.2–3.4.

The ANOVA results in Table 2 indicate that except for the variables of medical cost, environment, information channel, and age, the variables all show significant differences among different levels of hospitals (α = 0.05). For information channel groups, except for socio-demographics, the public channel scores of other variables are significantly higher than those for the private channel group; in particular, scores for getting information through public channels, hospital selection (loyalty), and satisfaction are significantly higher.

The results of multiple comparisons show that post reform, patients’ satisfaction in tertiary hospitals is significantly higher than that in primary and secondary hospitals, but no significant difference exists between primary and secondary hospitals. In addition, no significant difference exists in patient satisfaction with low-level hospitals between tertiary and other low-level hospitals, whereas patient satisfaction in secondary hospitals is significantly higher than that in primary hospitals.

The results for the two hospital selection (loyalty) variables are different: (1) although patients visiting a tertiary hospital have already chosen a high-level hospitals, their willingness to select a low-level hospitals is not significantly different from patients who attend other level hospitals; (2) those visiting secondary hospitals reported a higher score for this variable than those visiting primary hospitals; and (3) when a patients suffers a serious illness, their loyalty to their local low-level hospitals is significantly lower if their most recent hospital visit was to a primary hospital rather than a secondary or tertiary hospital, but no significant difference was found between the latter two.

Regarding the other exogenous latent variables, scores for trust, accessibility, capability, and convenience are significant higher for secondary hospitals compared with primary hospitals. In terms of socio-demographics variables, the education level of patients visiting tertiary hospitals (3.34 ± 1.05) is significantly higher than those of patients attending primary and secondary hospitals; no significant difference was found in education levels of patients visiting the latter two. Accordingly, the reform awareness of patients in tertiary hospitals is significantly higher than that among patients attending other levels of hospitals; however, their evaluation of reform-related policies like medical service prices and insurance reimbursement amounts is significantly lower.

### SEM estimation results of patient satisfaction

**Fig. 3:**
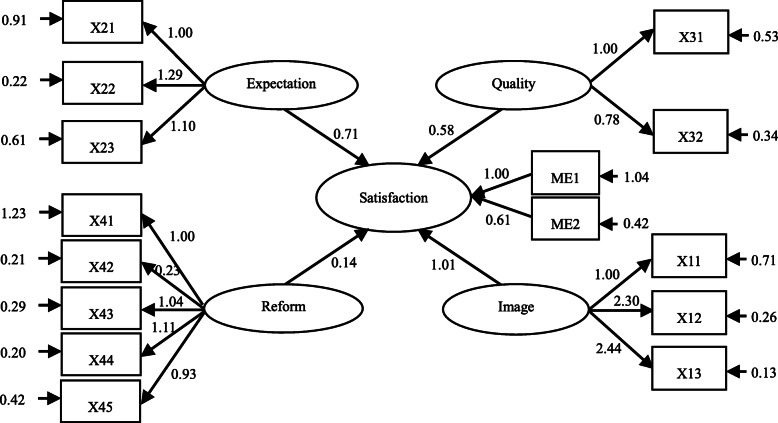
Patient satisfaction structural equation model path diagram. Source: The authors

The above discussion has offered some preliminary investigations into the effect of the descending resources reform on patient responses to the reform, but more evidence is needed to understand the marginal effect of different latent variables. Therefore, AMOS 21.0 software is used to test the theoretical model established in Fig. 1. This section reports the results of the patient satisfaction model. Following an iteration procedure using the bootstrap method, the resulting path diagram of the SEM model is shown in Fig. 3. Perceived quality, patient expectations, and hospital image all have a significant positive impact on patient satisfaction, and their normalized path coefficients of 0.577, 0.711, and 1.014, respectively, indicate that the ECSI model can better explain the factors influencing patient satisfaction. Overall, this model is significant. At the same time, the coefficient of the reform policy is 0.140, which confirms the reform’s positive effect on patient satisfaction.

The relationships between the variables and estimated coefficients are reported in Table 3. It can be seen that the effects of socio-demographics on patient satisfaction do not pass the significance test, but other latent variables are significant at α = 1%, which demonstrates the existence of a causal relationship between the variables. This in turn indicates that the structural equation model of patient satisfaction is appropriate. In this model, hospital image exerts the biggest influence on patient satisfaction, which in turn mainly originates from the trust in local low-level hospitals and the brand implantation of cooperative high-level hospitals.

**Table 3:** SEM estimation results of patient satisfaction model

Number	Relationship	Normalized path coefficient	Standard deviation	C.R. value	*P* values
1	Satisfaction←Perceived quality	0.577	0.051	11.246	***
2	Satisfaction←Patient expectation	0.711	0.071	10.051	***
3	Satisfaction←Hospital image	1.014	0.084	12.099	***
4	Satisfaction←Reform policy	0.140	0.040	3.521	***
5	Satisfaction←Socio-demographics	0.252	0.383	0.658	0.511

Note: *** indicates significance level of α =1%

Next, the confirmatory factor analysis method is used to conduct the single-factor structural validity analysis (Table 4). This analysis also serves as a reliability evaluation for the measurement model. The findings indicate that the coefficients for all latent variables except socio-demographics are significant at α = 1%. This demonstrates that the potential factor structure of the questionnaire items is reasonable. Several other findings can be drawn from the analysis. First, for the measurement variables of perceived quality, the factor loading coefficients of environment and convenience are quite close, indicating their important explanatory power on patients’ perceptions of quality. Second, for patient expectations, the factor loading coefficients of capability of low-level hospitals is the highest (=1.285), followed by medical cost and accessibility of descending doctors. Third, for hospital image, the factor loading coefficients of patients’ awareness of cooperative high-level hospitals and descending doctors reached 2.295 and 2.444, respectively, which are far higher than the score for the variable of trust (=1.000). Finally, for the latent variable of the reform policy, the factor loading coefficients of (differential) insurance imbursement levels and medical service prices are high (> 1), and the coefficient of tiered medical services reaches 0.933; these variables, together with reform awareness, thus have a strong explanatory power for the reform latent variable.

**Table 4:** Confirmatory factor analysis results of patient satisfaction model

Number	Relationship	Factor loading coefficient	standard deviation	C.R. value	*P* values
1	Reform satisfaction←Satisfaction	1.000	–	–	–
2	LLH satisfaction←Satisfaction	0.607	0.027	22.134	* * *
3	Convenience←Perceived quality	1.000	–	–	–
4	Environment←Perceived quality	1.004	0.066	15.197	* * *
5	Accessibility←Patient expectation	1.000	–	–	–
6	Capability←Patient expectation	1.285	0.090	14.200	* * *
7	Medical cost←Patient expectation	1.102	0.077	14.375	* * *
8	Trust←Hospital image	1.000	–	–	–
9	Awareness for high-level hospital←Hospital image	2.295	0.116	19.748	* * *
10	Awareness for descending doctors←Hospital image	2.444	0.125	19.536	* * *
11	Reform awareness← Reform policy	1.000	–	–	–
12	Information channel←Reform policy	0.228	0.023	9.728	* * *
13	Medical service price←Reform policy	1.039	0.060	17.257	* * *
14	Insurance imbursement←Reform policy	1.114	0.065	17.254	* * *
15	Tiered medical service←Reform policy	0.933	0.057	16.326	* * *
16	Gender←Socio-demographis	1.000	–	–	–
17	Age←Socio-demographis	16.821	6.333	2.656	0.008
18	Education level←Socio-demographis	13.768	4.853	2.837	0.005

Note: *** indicates significance level of α =1%

### SEM estimation results of patient hospital selection (loyalty)

**Fig. 4:**
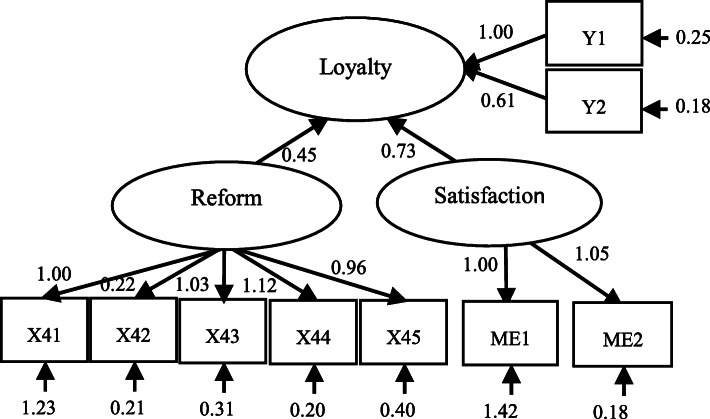
The path diagram of patient hospital selection (loyalty) . Source: The authors

Figure 4 shows the path diagram of the structural equation model for patient hospital selection (loyalty). The normalized path coefficients of the descending resources reform and patient satisfaction to loyalty are 0.450 and 0.731, respectively (Table 5). These two latent variables are significant at α = 1%, indicating that a causal relationship between variables can be established, and therefore, this structure equation model of patient hospital selection is appropriate. It can be seen that the impact of patient satisfaction on loyalty is greater than that of the reform policy, which indicates patients’ LLH choice is more affected by their own evaluations of the reform and local low-level hospitals.

**Table 5:** SEM estimation results of patient hospital selection (loyalty) model

Number	Relationship	Normalized path coefficient	Standard deviation	C.R. value	*P* values
1	Loyalty←Satisfaction	0.731	0.052	14.041	* * *
2	Loyalty←Reform policy	0.450	0.043	10.570	* * *
3	Loyalty←Socio-demographics	0.034	0.380	0.091	0.928

Note: *** indicates significance level of α =1%

The confirmatory factor analysis results for the hospital selection (loyalty) model are reported in Table 6. The coefficients of different socio-demographics are still significant at α = 1%, but this latent variable itself does not have a significant impact on loyalty according to the results reported in Table 5. The two measurement variables of loyalty have close factor loading coefficients (1.000 and 0.963), showing their strong explanatory power. Satisfaction with the reform and LLHs also have similar factor loading coefficients (1.000 and 1.054).

**Table 6:** Confirmatory factor analysis results of patient hospital selection (loyalty) model

Number	Relationship	Normalized path coefficient	Standard deviation	C.R. value	*P* values
1	Y1 ← loyalty	1.000	–	–	–
2	Y2 ← loyalty	0.963	0.027	35.719	* * *
3	Reform satisfaction←Satisfaction	1.000	–	–	–
4	LLH satisfaction←Satisfaction	1.054	0.075	14.000	* * *
5	Reform awareness←Reform policy	1.000	–	–	–
6	Information channel←Reform policy	0.219	0.023	9.7375	* * *
7	Medical service price ←Reform policy	1.028	0.060	17.165	* * *
8	Insurance imbursement←Reform policy	1.119	0.064	17.399	* * *
9	Tiered medical service←Reform policy	0.963	0.058	16.552	* * *
10	Gender←Socio-demographics	1.000	–	–	–
11	Age←Socio-demographics	16.773	6.378	2.630	0.009
12	Education level←Socio-demographics	13.699	4.823	2.840	0.005

Note: *** indicates significance level of α =1%

In terms of the measurement variables of the reform policy, the factor loading coefficients of (differential) insurance imbursement levels and medical service prices are still greater than 1 (=1.119 and 1.028), which shows their important impact on perceptions of the descending resources reform. The factor loading coefficients of reform awareness and tiered medical services also reached 1.000 and 0.933, showing their explanatory power of acceptance of the reform.

### Robustness test

In order to test the robustness of the above full-sample results, we use different-level hospital subsamples to perform empirical analyses. The sample sizes of different level hospitals meet the requirements of carrying out SEM model estimation.

Table 7 reports the estimation results of different subsamples. It can be seen that, firstly, the impacts of perceived quality, patient expectations, and hospital image on patient satisfaction are confirmed to be positive again for the tertiary and secondary hospital subsamples at the 1% significance level. However, for the primary hospital subsample, the impact of patient expectations is slightly insignificant (*P* = 0.072). Second, for tertiary and secondary hospitals subsamples, the positive impact of the reform latent variable on patient satisfaction is significant at α = 5%, although it is insignificant for the primary hospital subsample. This makes sense because doctors descending from high-level hospitals in Zhejiang Province only descend to secondary hospitals, so this reform latent variable’s impact on primary hospitals is insignificant. Third, socio-demographics still have an insignificant effect on patient satisfaction. Finally, subsample results for patient hospital selection (loyalty) show strong robustness in terms of satisfaction and the reform’s positive effect, as well as the insignificant effect of socio-demographic variables. Thus, it can be concluded that the subsample empirical analysis supports the estimation results of the full-sample SEM model.

**Table 7:** Subsample estimation results of patient satisfaction and hospital selection (loyalty) models

Number	Relationship	Subsample	Normalized path coefficient	Standard deviation	C.R. value	*P* values
1	Satisfaction← Perceived quality	Tertiary	0.335	0.087	3.836	* * *
		Secondary	0.313	0.039	8.085	* * *
		Primary	0.488	0.119	4.089	* * *
2	Satisfaction← Patient expectation	Tertiary	0.271	0.100	2.708	0.007
		Secondary	0.502	0.058	8.603	* * *
		Primary	0.258	0.144	1.799	0.072
3	Satisfaction← Hospital image	Tertiary	0.456	0.080	5.718	* * *
		Secondary	0.571	0.047	12.051	* * *
		Primary	1.120	0.305	3.667	* * *
4	Satisfaction← Reform policy	Tertiary	0.267	0.070	3.807	* * *
		Secondary	0.051	0.026	2.004	0.045
		Primary	0.101	0.116	0.870	0.385
5	Satisfaction← Socio-demographics	Tertiary	0.073	0.406	0.179	0.858
		Secondary	0.099	0.276	0.358	0.720
		Primary	0.677	1.309	0.517	0.605
6	Loyalty← Satisfaction	Tertiary	0.540	0.128	4.214	* * *
		Secondary	0.615	0.058	10.632	* * *
		Primary	0.885	0.199	4.448	* * *
7	Loyalty← Reform policy	Tertiary	0.599	0.120	5.000	* * *
		Secondary	0.379	0.040	9.372	* * *
		Primary	0.784	0.335	2.339	0.019
8	Loyalty← Socio-demographics	Tertiary	1.344	1.147	1.172	0.241
		Secondary	0.157	0.415	0.378	0.706
		Primary	1.874	8.400	0.223	0.823

Note: *** indicates significance level of α =1%

### Mediation effects estimation result

In order to estimate the impact of the reform latent variable on hospital selection (loyalty) through satisfaction, we use the method shown in Fig. 2 to perform the test the mediating effect (Table 8). It can be seen that *β*^*’*^_*c*_ is significant but its absolute value is less than *β*_*c*_ in the full-sample estimation, which confirms the existence of a partial mediating effect. This finding indicates that the reform latent variable has an indirect impact on hospital choice behavior through satisfaction, but it also directly affects hospital choice. In order to verify the robustness of this result, we also perform hospital subsample estimations, the results of which show that heterogenous results exist for different hospital subsamples. The results for the tertiary and secondary hospitals subsamples show a complete mediating effect, which is consistent with the previous literature on the impact of satisfaction upon loyalty implied in ECSI model ^[18.19]^; however, no mediating effect is found for the primary hospital subsample, which may be related to the fact that the descending resources reform in Zhejiang mainly involves secondary and tertiary hospitals but not primary hospitals.

**Table 8:** Mediation effects estimation result of reform latent variable

Exogenous variable: Reform; Mediating variable: Satisfaction; Exogenous variable: Hospital selection (Loyalty)						
Sample		*β*_*a*_	*β*_*b*_	*β*_*c*_	*β*^*’*^_*c*_	Conclusion
Full-sample		0.855***	1.579***	0.752***	−0.683*	Partial mediating effect
Hospital subsample	Tertiary	0.758***	1.148**	0.470***	−0.144	Complete mediating effect
	Secondary	0.846***	1.439***	0.760***	−0.535	Complete mediating effect
	Primary	1.010***	1.439	0.778***	0.945	No mediating effect

Note: ***and** indicate significance level of α =1 and 5%, respectively

## Discussion

China’s descending resources reform offers a unique approach to overcoming the uneven allocation of health resources. It works by utilizing the dominant role of the public hospital system in the Chinese health market compared with other developing countries. Using questionnaire data, the expanded ECSI model, and the structure equation model, we found that the descending resources reform had a significantly positive impact on patients’ satisfaction and their selection of local low-level hospitals. It was further found that the measurement variables of perceived quality, patient expectations, and hospital image can also explain patient behavior. ANOVA and multiple comparison techniques demonstrated that significant differences exist among patients at different levels of hospitals.

Thus, it is found that the descending resources reform contributed to the improvement of convenience, capabilities, and the environment of local low-level hospitals, offering evidence of the effect of this reform from the patients’ perspective. This paper’s SEM estimations also confirm the significantly positive impacts of perceived quality, patient expectations, and hospital image on patient satisfaction. Among these, the treatment/diagnosis capabilities of low-level hospitals were found to have the strongest explanatory power for patient expectations, indicating that LLH capabilities are a top factor shaping patients’ expectations, and this issue is also the core focus of the descending resources reform.

Among the different latent variables, hospital image exerts the greatest influence. Of the three measurement variables, i.e., trust in low-level hospitals and awareness of cooperative high-level hospitals and descending doctors, the confirmatory factor analysis suggested that the latter two have higher explanatory power than trust in low-level hospitals. This result shows that embedding the brand image of high-level hospitals and doctors into low-level hospitals is an effective way to improve the image of low-level hospitals, and the role of brand implantation is greater than that of the image (trust) of low-level hospitals from the patient perspective. Furthermore, improving the image of low-level hospitals can also significantly contribute to higher patient satisfaction and loyalty to low-level hospitals, which is fully consistent with the goal of the descending resources reform.

This study also confirmed the significantly positive impact of the reform and satisfaction on patients’ hospital selection (loyalty) of low-level hospitals and the existence of mediating effects of satisfaction. Previous literature has found that medical service quality affects patient satisfaction, which in turn affects their behavioral choices [37]; high service satisfaction has a significantly positive impact on customer loyalty [38]. The empirical results on the mediating effect confirm that in secondary and tertiary hospitals, the reform latent variables affect patients’ hospital choice behavior through the mediating factor of satisfaction. However, such effects disappear for primary hospitals, a difference that highlights the reality that descending resources reform in Zhejiang focused more on secondary and tertiary hospitals. This paper provides new evidence based on patient behavior in the context of China’s latest healthcare reform, and evidence for the appropriation of expanded ECSI model used in this paper.

Zhejiang’s reform is characterized by descending doctors from high-level hospitals to low-level hospitals, together with important supporting policies like medical service prices, differential insurance imbursement levels, and tiered medical services. These policies all received higher scores from patients, with patients visiting a secondary hospital evaluating them significantly higher than those visiting a tertiary hospital, again indicating the focus and main beneficiaries of this reform being secondary hospitals. Meanwhile, such polices contribute to explaining the reform’s latent variables well and have significantly positive effects on patient satisfaction and loyalty. These results suggest that the above supporting policies can help reduce the burden of medical costs incurred by patients in low-level hospitals, and their satisfaction with and loyalty to low-level hospitals can be improved via financial incentives [39].

The survey results also show the coexistence of low reform satisfaction and high satisfaction with local low-level hospitals, where patients visiting tertiary hospitals have higher reform satisfaction, whereas those visiting secondary hospitals have higher satisfaction with local low-level hospitals than those visiting primary hospitals. Correspondingly, the reform awareness of patients visiting tertiary hospitals is also significantly higher than those visiting other levels of hospitals. This could reflect the fact that one of the reform’s main goals was to reduce congestion in high-level hospitals by reallocating health resources to different levels of hospitals. Thus, patients visiting high-level hospitals should have a real understanding of the reform’s effects, benefit from the reform, and have higher satisfaction with reform. Meanwhile, in this reform, descending doctors mainly flow into secondary hospitals, which receive the greatest amount of resources inflow and capability improvements, thus contributing to their patients’ satisfaction compared with those visiting primary hospitals.

It is worthy pointing out the importance of information channels on the reform’s effect. The ANOVA results indicate that reform awareness and patient behavior are related to the information channel. Information access via public channels can avoid information transmission distortion and thus help promote a positive response from patients. Although according to the SEM estimation, information channels do not have a strong explanatory power for the reform latent variable, the ANOVA results showed that scores of perceived quality, patient expectation, hospital image, satisfaction and hospital selection (loyalty), are all significantly higher for the public channel subsample than the private channel subsample, indicating that a more unbiased information supply and effective transmission are essential in enhancing patients’ positive responses and reshaping their hospital choices.

## Conclusion

China’s reforms carried out since 2003 have offered evidence that traditional approaches focusing on higher public health expenditure and expansion of medical insurance coverage are inadequate in correcting the uneven allocation of health resources. The descending resources reform launched in 2013 is an attempt to improve the capability of low-level hospitals and re-attract patients by using human capital spillovers of doctors descending from high-level hospitals together with brand implantation of these high-level hospitals. Using questionnaire survey data collected from patients in Zhejiang Province, this paper provides supporting empirical evidence of the reform’s impact on patient behavior. The results indicate that the reform has been effective in improving the capabilities of low-level hospitals, and brand implantation of high-level hospitals shows strong explanatory power. The findings also suggest that policymakers could pay more attention to the importance of information channels in impacting patient awareness, responses, and hospital selection.

For developing countries where public hospitals play a dominant healthcare role, China’s reform offers a distinct and valuable approach to correcting the uneven allocation of health resources. The approach indicates that greater investment and demand-side reforms might not necessarily incentivize patients to respond as the government intends. In addition to affordability, building the capabilities of hospitals in rural/remote areas remains a key factor, which can be done by cooperation between high- and low-level hospitals, human capital spillovers, and brand implantation. Meanwhile, some demand-side policies like differential medical insurance and price regulations could also create incentives for patients to reshape their hospital choices so as to resolve the existing structural congestion or uneven allocation problems. In addition, access to full and unbiased information is of great importance in realizing the full effect of these reforms. Therefore, the findings of this paper highlight that the government should pay attention to public information channels in improving the effect of healthcare reforms. Besides, after the data collection of this paper, the outbreak of COVID-19 pandemic encourages patients to choose local low-level hospitals more so as to avoid travelling and infection risk to high-level hospitals, so this phenomenon could enhance the existing effect of the above reform on patients’ choice to low-level hospitals, and it does not harm the applicability and usefulness of our findings.

## Data Availability

The datasets during the current study available from the corresponding author on reasonable request.

## Abbreviations

ECSI: The European consumer satisfaction index model
ANOVA: Analysis of variance
SEM: Structure equation model
SARS: Severe acute respiratory syndrome
KMO: Kaiser-Meyer-Olkin test
OLS: Ordinary least square
OLM: Ordered logit model

## Footnotes

1 The authors thank one anonymous reviewer’s very helpful comment for this term difference. The traditional literature usually discusses individual-level response on health service through indicators of the physical environment, patient-friendly environment, response capacity, communication, privacy, and security. Some other factors, like knowledge of history, monitoring of health problems, and information, are also discussed.
